# Case Report: Surgical timing for Blount’s disease: a case report and systematic review

**DOI:** 10.3389/fendo.2025.1547679

**Published:** 2025-04-09

**Authors:** Bing Wang, Zukang Miao, Xiuchun Yu, Ke Zhou, Ning Liu, Kai Zhai, Shu Zheng, Haining Sun

**Affiliations:** Department of Orthopedics, 960th Hospital of the People’s Liberation Army, Jinan, China

**Keywords:** blount’s disease, operation timing, treatment, osteotomy, early-onset; late-onset

## Abstract

**Background:**

Currently, Blount’s disease is treated in a variety of ways, but the optimal timing of treatment and the choice of optimal treatment regimen have yet to be determined. We report a case of a patient who failed multiple surgical treatments and underwent 3D-printed osteotomy guide-assisted proximal tibial orthopedic external fixation in adulthood to restore normal lower limb mechanical axis and suggest optimal treatment modalities in light of the systematic literature.

**Methods:**

A case of Blount’s disease patient who was misdiagnosed and missed and underwent multiple surgical treatments was retrospectively studied. According to the PRISMA statement, a systematic review of electronic databases such as PubMed, Embase, and Web of Science was conducted to explore the optimal timing of surgery for Blount’s disease from 2010.

**Results:**

A boy born in 2001 was found to have a varus deformity in his left knee joint at the age of 2 years, which was not diagnosed. At the age of 7 years, he was diagnosed with Blount’s disease and underwent multiple surgeries over the following years, all of which resulted in recurrences. At the age of 21 years, he underwent high osteotomy and external fixation of the proximal left tibia using a 3D-printed guide plate in our hospital. At present, the external fixation has been taken out, and the lower limb force line has recovered well. The timing and choice of treatment for Blount’s disease are important for the patient’s prognosis. The systematic review analyzed a total of 23 studies with a combined sample size of 679 cases, it provides recommendations for treatment strategies based on patient age.

**Conclusion:**

The patient’s age and degree of deformity are key in determining the timing and treatment plan. For patients with early-onset, who are under four years old, they may begin with a conservative treatment strategy, moving to a timely osteotomy if the initial approach proves ineffective. For patients with late-onset, 4-10 years old, there are no recommendations for definitive treatment at this time. Patients over 10 years old should have their bone age and growth potential evaluated, with epiphysiodesis recommended for those with a growth potential greater than 2 years and osteotomy recommended for those with less than 2 years to achieve a complete correction of the deformity.

## Introduction

Blount’s disease is a spontaneous condition that causes an abnormal unilateral or bilateral growth of the medial proximal tibia, leading to severe tibial varus deformity. The disease is classified into two main categories based on age at onset: Early-onset, detected before the age of four years, and late-onset, detected after this age (1). A more specific classification further segments the late-onset category into early-onset/infantile (less than 4 years old), juvenile (4–10 years old), and adolescent (more than 10 years old) (2). Prognosis, intervention timing, and treatment modalities for Blount’s disease can vary significantly, as evidenced by domestic and international studies. Thus, based on the diagnosis, treatment process, and prognosis of a Blount’s disease patient treated at our hospital, and in combination with relevant literature, we aimed to discuss the most recent strategies for the timing of Blount’s disease treatment.

## Case presentation

**Figure 1:**
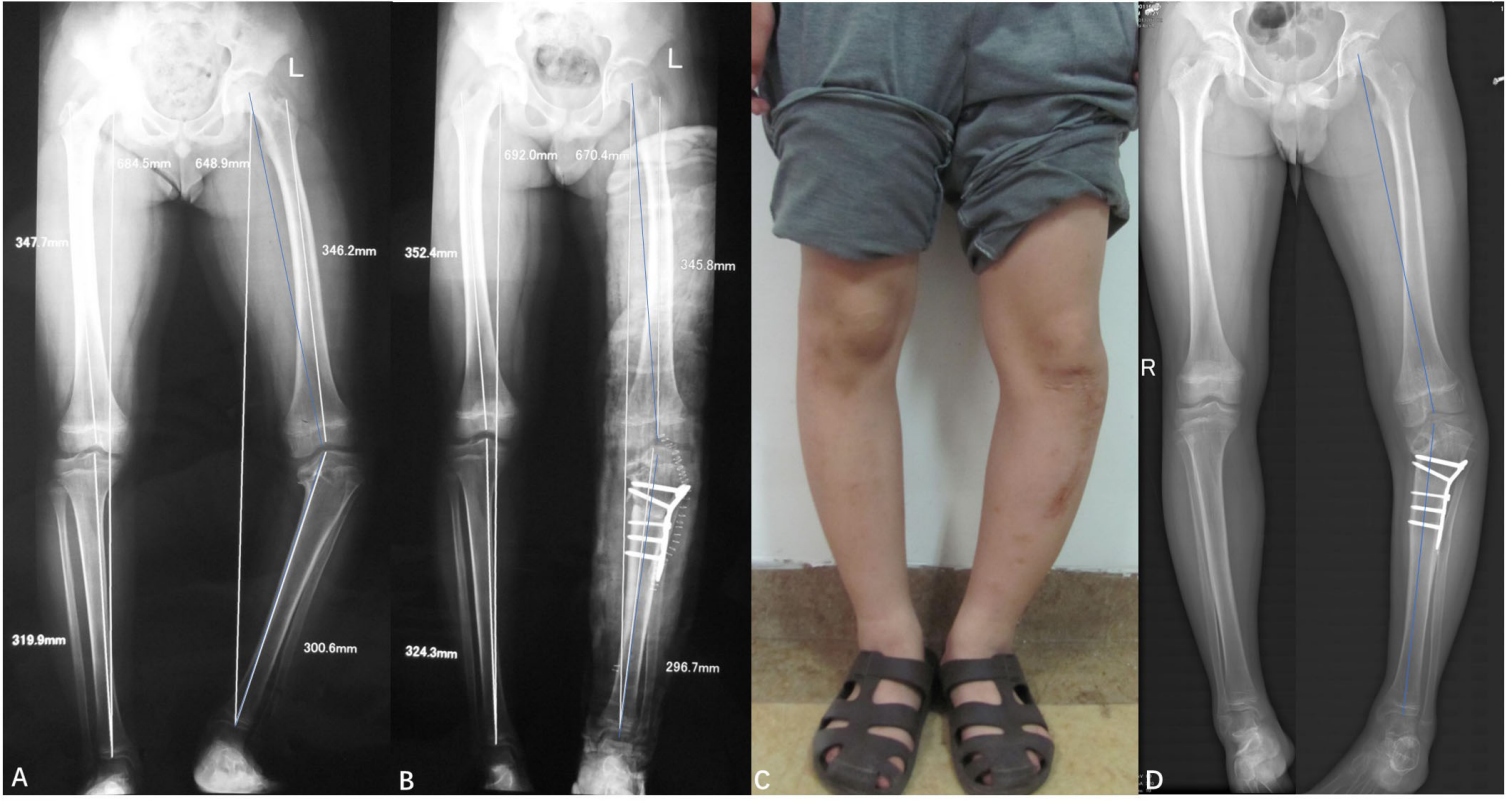
**(A)** Full-length X-ray of both lower limbs (October 2011, preoperative): Demonstrates varus deformity of the left tibia, wedge-shaped thinning of the medial epiphysis of the proximal tibia, and uneven osteogenesis of the proximal metaphysis. The MAA is 148°. **(B)** (December 2011, post-secondary surgery): Shows solid internal fixation in an optimal position, MAA: 170°. **(C)** Photograph of the left lower leg (July 2013, pre-internal fixation removal): Prominent varus deformity with a well-healed 15 cm incision on the anterior aspect of the tibia. **(D)** Full-length X-ray of both lower limbs (July 2013, pre-internal fixation removal): Reveals good healing of the osteotomy end and stable internal fixation, MAA: 164°.

A male patient, born in March 2001, presented with a varus deformity in his left knee joint that has existed since the age of two. No genetic disease in the patient’s family. Initial consultation at a local healthcare facility raised suspicions of rickets, leading to a three-year regimen of intermittent calcium and vitamin D tablets, not treated with conservative treatment such as knee ankle foot orthosis(KAFO). However, this treatment failed to substantially alleviate his symptoms, and the varus deformity progressively worsened. In May 2008, at the age of seven, the patient underwent further diagnostic imaging at a second medical institution. The clinical presentation and subsequent diagnostic results confirmed a diagnosis of Blount’s disease. Following appropriate surgical considerations, the patient underwent high osteotomy and external fixation of the left tibia. In December 2011, he received a navigational surgery at another hospital to open the left proximal tibial medial epiphysis, followed by high proximal tibial osteotomy and internal fixation (**Figures 1A, B**). Post-operatively, the leg was immobilized in plaster for two months and weight-bearing was gradually introduced. Despite these measures, the varus deformity persisted. The patient presented to our institution in July 2013 for internal fixation (**Figures 1C, D**). X-ray shows medial step-like changes in the proximal tibial metaphysis. Blood biochemical parameters were normal, physiologic knee inversion and rickets were ruled out, and the diagnosis of blount’s disease was once again made definitively. Upon removal of the fixation, the varus deformity of his knee deteriorated further. Given this history of left tibial varus deformity lasting over 14 years and recent onset left knee joint pain of one-month duration, our surgical team performed high osteotomy and external fixation of the proximal left tibia using a 3D printed guide plate on April 8, 2022. Follow-up appointments showed promising prognosis, and the external fixator was successfully removed on January 31, 2023.

**Figure 2:**
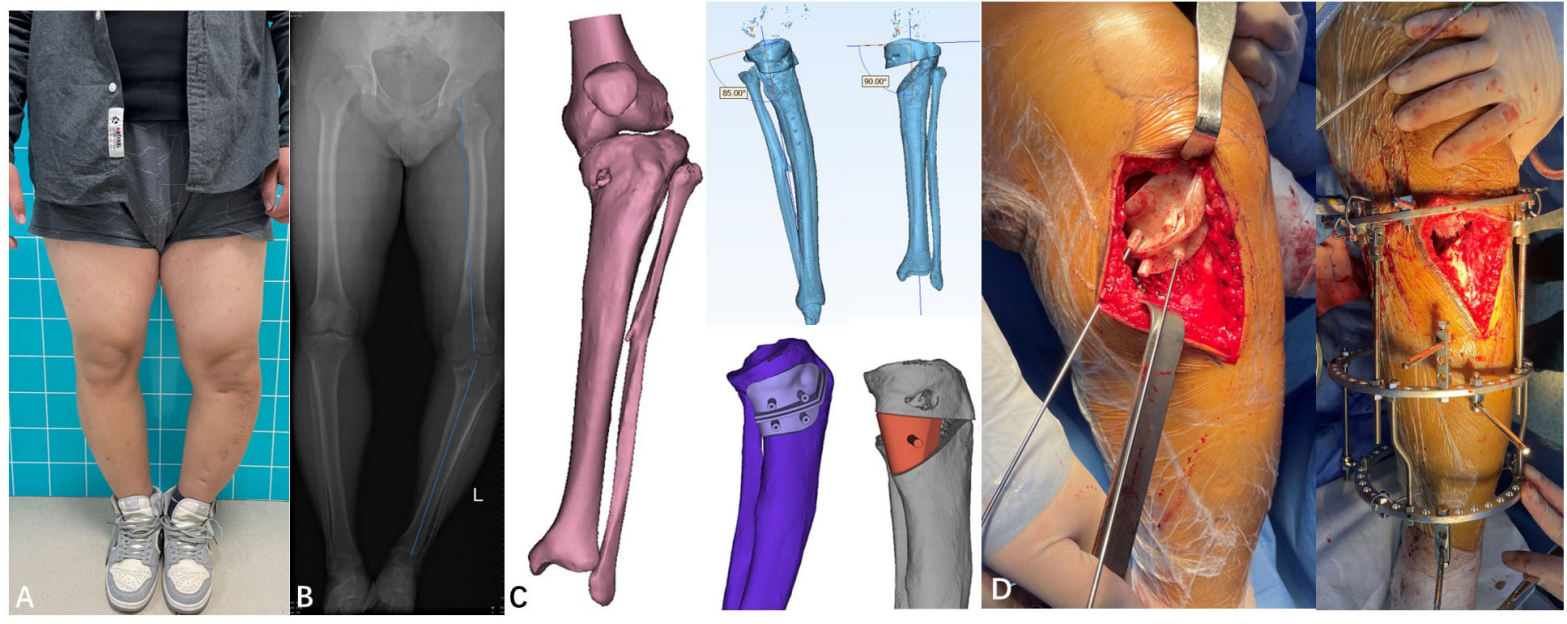
**(A)** Photograph (March 2022, prior to third surgery). **(B)** Full-length X-ray of both lower limbs (March 2022, prior to third surgery): Shows left knee joint varus deformity, uneven tibial plateau with multiple cystic low-density shadows, and disorder in the trabecular structure, MAA: 158°. **(C)** Preoperative 3D-printed osteotomy design plan, Intraoperative image showing a 3D-printed osteotomy guide plate assisting in the osteotomy. **(D)** Simulation of the preoperative osteotomy angle adjustment, Intraoperative image showing osteotomy and fixation using an external fixator.

Upon admission on March 29, 2022, physical examination revealed the following parameters: height, 178 cm; weight, 104 kg; and a body mass index (BMI) of 32.82 kg/m2. Notable findings included a claudicating gait and a varus deformity of the left knee. There was a visible surgical scar, measuring 20 cm in length, proximal to the left tibia, with no associated tenderness around the incision or the left knee joint. The left lower limb was 3 cm shorter than the right. Both knee joints exhibited a normal range of motion, and muscle strength, tension, and skin sensation were normal in both lower limbs. Physiological reflexes were present and no pathological reflexes were noted (**Figure 2A**). Laboratory tests revealed: white blood cell (WBC) count, 6.44 x 109/L; red blood cell (RBC) count, 5.69 x 109/L; Hemoglobin (Hb), 165 g/L; Platelet count (Plt), 217 x 109/L; C-reactive protein (CRP), 16.5 mg/L; and uric acid (UA), 466 μmol/L. Full-length X-rays of both lower limbs demonstrated leg length discrepancy, with the right lower limb being 2.3 cm longer than the left. There was malformation in the left lower limb, with irregular bone structure in the proximal tibia, multiple cystic low-density areas, and misalignment of the femur and tibia. The pelvis appeared slightly tilted to the left (**Figure 2B**). A full-length CT scan of both lower limbs confirmed the irregular proximal bone structure of the left tibia, the presence of multiple cystic lucencies internally, and disordered trabecular bone structure. The medial condyle of the tibia was undersized, and there was poor alignment and asymmetry of the left knee joint space. The left fibula exhibited a localized bony protrusion and an enlarged mid-section, with no other evident abnormalities.

**Figure 3:**
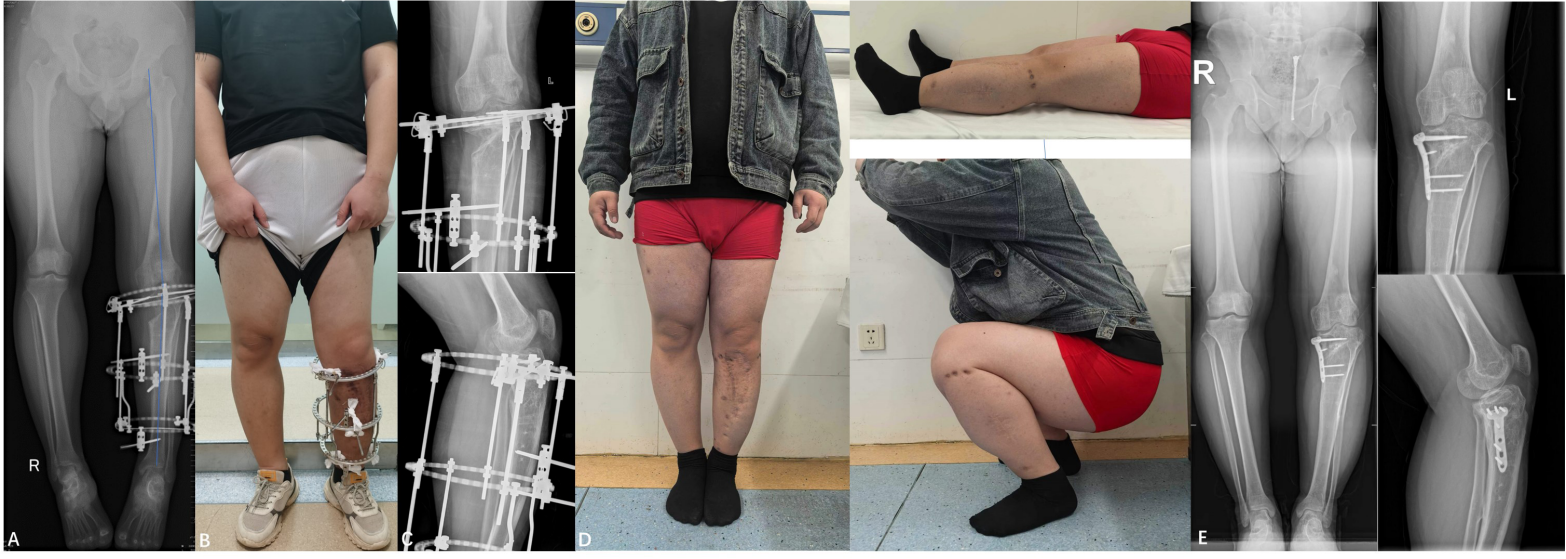
**(A)** Full-length X-ray of both lower limbs (April 2022, post-third surgery): Features the external fixator in a good position, normal force lines of both lower limbs, aligned osteotomy ends, the defect filled with high-density bone, and the left limb being shorter than the right by 1 cm, MAA: 176°. **(B)** Photograph (June 2022, post-third surgery): Shows the lower limb deformity completely corrected and fixed with an external fixator, with no obvious discomfort observed. **(C)** X-ray of left tibia and fibula (January 2023, post-third surgery): Demonstrates a medial bone defect in the proximal tibia, good alignment of the osteotomy ends, and a well-positioned external fixator ten months following surgery. **(D)** Photograph (February 2023): Image captured after the removal of the external fixator. **(E)** X-ray(February 2023):MAA:174°.

The proposed diagnoses included (1): left tibial varus deformity; (2) Blount’s disease; (3) postoperative tibial osteotomy. Noteworthy physical examination results revealed significant deformity on the affected side with a femorotibial mechanical axis angle (MAA) of 158°. Considering the patient’s multiple anterior and posterior surgeries, heavy deformity, and poor bone quality, the design of osteotomy guides using 3D printing technology was able to minimize surgical trauma and achieve precision in osteotomy. A 3D-printed osteotomy guide plate was fabricated based on comprehensive CT scans of both lower limbs. The distraction angle was set to approximately 35 degrees (**Figure 2C**). Using C-arm fluoroscopy, the mechanical axis of the lower limbs was restored. Subsequently, a circular external fixator was applied, an allogenic bone graft was inserted at the osteotomy site, and a drainage tube was placed *in situ* (**Figure 2D**). The procedure was executed and concluded successfully. Postoperative management included standard anti-inflammatory, analgesic, and antithrombotic regimens. The surgical incision healed well, and sutures were removed without complications (**Figures 3A–C**). Regular follow-ups were scheduled every three months post-operation. The external fixator was removed and installed internal fixation to prevent fracture in January 2023.The strength line of the affected limb basically returned to normal (MAA: 174°). The physicians and the patient’s family observed that the walking posture was significantly improved. The patient said there is no obvious pain and discomfort during walking. and basically returned to normal study and life without assistance.Everyone was very satisfied with the results of the surgery. On March 10, 2025, the patient reported no significant discomfort and had resumed normal exercise and life with no significant deformity of the affected limb(**Figures 3D, E**).

## Systematic review

### Literature search

A search of PubMed, Embase, and Web of Science electronic databases was conducted to screen for Blount’s disease. The language of publications was limited to English. Publication dates were after 2010. The search terms were (“blount’s disease” OR “blount disease” OR “congenital knee inversion deformity”) AND (“treatment” OR “surgery” OR “postoperative recurrence”) by title/abstract. An additional search was performed for references cited in all relevant articles.

### Data extraction

Data extracted from eligible papers included baseline characteristics, orthopaedic investigations, imaging findings, treatment options and clinical follow-up results. Data collection was conducted independently by two reviewers using a standard form. If disagreements persisted, arbitration was conducted by a third review author.

### Literature selection and quality assessment

**Figure 4:**
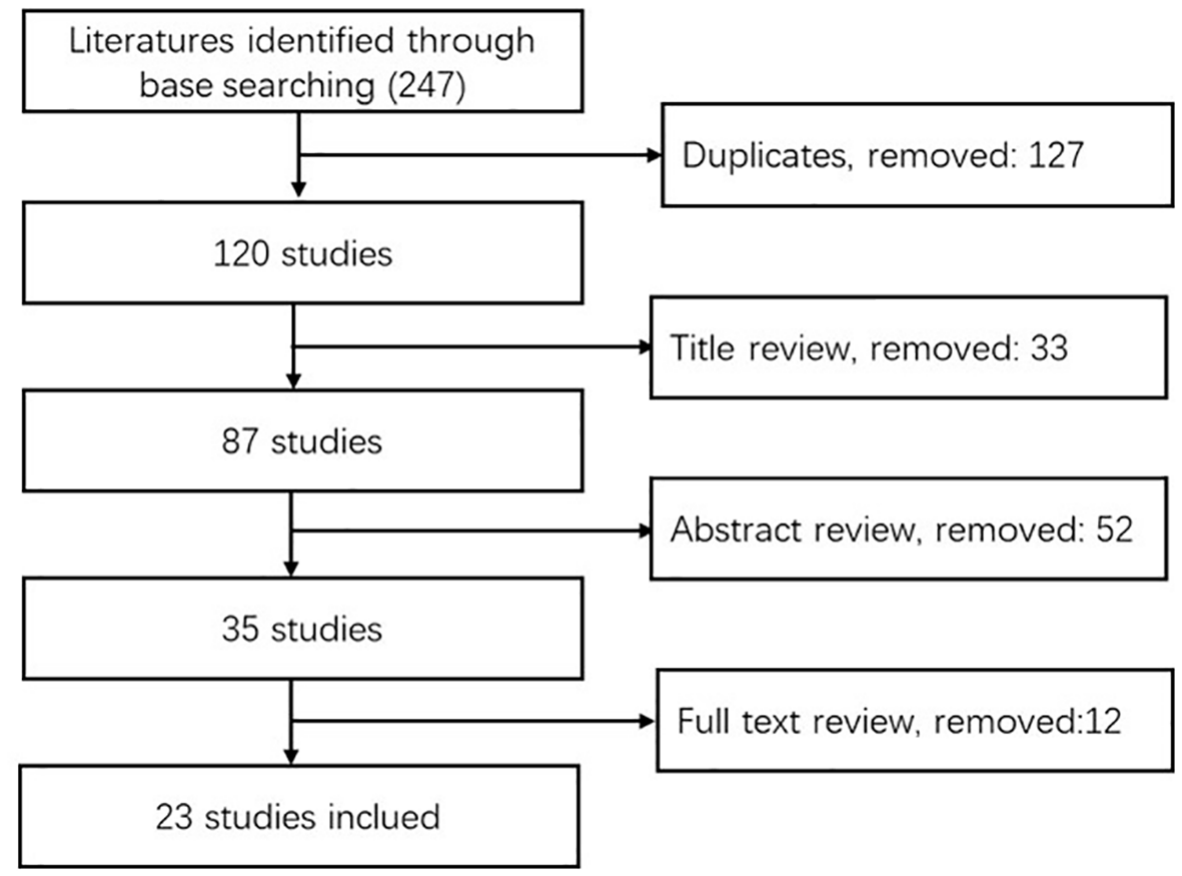
PRISMA Flow-chart.

The initial search revealed 247 studies. After removal of duplicates, exclusion by title and abstract, and exclusion by full text, a total of 23 documents were finally included (**Figure 4**). All of the literature was assessed by the JBI quality assessment tool to be of adequate quality level.

### Data analysis

We reviewed the Blount’s disease previously reported (**Table 1**) (3–25). The selection consisted of 23 articles, including 18 retrospective studies, 4 case reports, and 1 prospective study, encompassing a total of 679 cases: infantile or early-onset, with 358 cases at Langenskiöld stages II through VI; and late-onset, with 210 cases, distributed among juveniles (12 cases), adolescents (70 cases), unclassified (128 cases), and those with unclear categorization (111 cases). The age range was 1.4 to 17 years. Treatments involved conservative management, osteotomy, hemiepiphysiodesis, epiphysiodesis, hardware removal, internal/external fixation and hemiplateau elevation.

**Table 1:** Cases in the literature.

NO.	Study	Country	Design	Limbs	Age (year)	Typing	Langenskiö-ld stage	Treatment	Fllowing (year)	Conclusion
1	Chandankere etal 2023 (3)	India	Retrospective	8	9.4 (8–11)	Infantile	IV-VI	Medial hemi-plateau elevation and metaphyseal osteotomy	3-8	It is crucial to achieve proper correction of all deformity components during surgery, with epiphysiodesis on the lateral side to prevent further recurrence.
2	Miraj etal 2023 (4)	Indonesia	Retrospective	25	8.8 (6-14)	infantile	III-VI	Acute correction with simultaneous hemiepiphysiodesis of lateral proximal tibia	3	Acute correction with simultaneous hemiepiphysiodesis of lateral proximal tibia physis is an effective technique to prevent deformity recurrence
3	Mare etal 2022 (5)	South Africa	Retrospective	25	12.5 (7-17)	15 Early-onset 10 Late-onset		Gradual correction with a computer-assisted hexapod external fixator	0.5-5.6	Recurrence remains a concern if correction is performed before skeletal maturity
4	Assan etal 2021 (6)	Benin	Retrospective	24	3-12	infantile		Guided growth or Tibial Osteotomy	>1	Guided growth appears to be the best treatment for early stage of disease in squelletically immature patients.
5	Baraka etal 2021 (7)	Egypt	Retrospective	21	10.3 (8.2 - 13.6)	infantile	V	Single-stage medial plateau elevation and metaphyseal osteotomies	5.1 (3.2-8.3)	It significantly improved the clinical and radiographic parameters and PODCI score in advanced infantile Blount’s disease and precluded the use of external immobilization, with no evidence of deformity recurrence.
6	Nada etal 2021 (8)	Egypt	Prospective	11	13.5 (11.5 - 15)	infantile	IV,V	Acute Correction and Plate Fixation	2.2 ± 0.5	This single-stage double osteotomy technique has been shown to be successful for this sample of patients and yielded acceptable clinical and radiological outcomes, without significant major complications.
7	Zein etal 2021 (9)	Egypt	Retrospective	32	13-22	Adolescent		Minimally invasive osteotomy and simple circular fixation	>2	Acute correction and simple circular frame fixation is an excellent treatment choice for cases of late-onset tibia vara, especially in severe deformities
8	Griswold etal 2020 (10)	USA	Retrospective	17	3.2 ± 1.4	infantile		Guided growth	≤2: 4.11 ± 2.2 ≥ 3: 4.81 ± 1.8	Caution should be used with the consideration of guided growth forchildren presenting with Langenskiold stage ≥3 due to high rate of recurrent deformity and need for subsequent surgeries
9	Musikachart etal 2020 (11)	Thailand	Retrospective	72	2.9 (2.2–3.9)	Infantile	II	Dome or wedge-shaped proximal tibial osteotomy	4.77 ± 2.78	Don’t demonstrate significant differences in the posterior tibial slope (PTS) angle.
10	Marine etal 2020 (12)	USA	Case report	1	5 years 4 months	Infantile	IV	Lateral proximal physeal tethering and medial hemiplateau elevation osteotomy	2	These procedures allow for correction of alignment while preserving growth potential.
11	Danino etal 2020 (13)	Israel	Retrospective	55	9.5 (1.58 -14.83)	11 Infantile, 12 Juvenile, 32 Adolescent		Temporal hemiepiphysiodesis	2(0.3- 3.5)	Adolescent Blount has the best chance of achieving full correction while same treatment is less effective in infantile Blount.
12	Miraj etal 2019 (14)	Indonesia	Retrospective	27	7.8	Infantile	III-VI	Step Cut “V” osteotomy	1	The method can produce an accurate correction, high union rate and early weight bearing with no complication as a result that would be achieved at the end of treatment.
13	Jain etal 2019 (15)	USA	Retrospective	61	9.6 (3.3 -14.8)	19 Early-Onset; 42 Late-Onset		Tension Band Plate (TBP)-guided Hemiepiphysiodesis	3.1 (0.6-9.9)	TBP still needs to be proven but 41% surgical failure rate in our study does not restrict the use of TBP as first choice implant in Blount disease.
14	Terjesen etal 2018 (16)	Norway	Case report	1	13	Unknown		Intraepiphyseal osteotomy with elevation of the medial plateau of the tibia	65	The method can be recommended in older children with pronounced varus deformity.
15	Tsibidakis etal 2017 (17)	Italy	Retrospective	24	7.5 (3–14)	Unknown		Proximal tibial osteotomy and Taylor Spatial Frame	3-6	TSF allows safe gradual correction and is an accurate and well-tolerated method for the treatment of the Blount disease at any age
16	Shawn etal 2015 (18)	USA	Retrospective	38	11.5 (7.5 ~ 15.2)	Late-Onset		Staple or Plate Hemiepiphysiodesis	3 (1.1 -5.3)	There was no difference in success between implant types and hemiepiphysiodesis is not recommended for morbidly obese patients with severe deformity
17	Liu etal 2015 (19)	China	Retrospective	7	1.4 -5.2	Infantile	II-IV	Femur,tibia and fibula osteotomies	3 -16	Femur, tibia and fibula osteotomies are useful for correction of Blount’s disease. The complication is less and can get satisfied result.
18	Sabharwal etal 2014 (20)	USA	Retrospective	70	Early-Onset 7.1 (5.3-8.9); Late-Onset 12.8 (12.0-13.5)	33 Early-onset 37 Late-onset	II	Osteotomy; Hemiepiphyseodesis; Epiphyseodesis; Hardware removal	4 (3.25-4.7)	The BMI of the majority of children with Blount’s disease increased over time and obesity needed to deal with.
19	Eamsobhana etal 2014 (21)	Thailand	Retrospective	65	2.8 (2.5–3.3)	Infantile	II	Dome osteotomy	3	There was no relationship between the amount of overcorrection to a valgus position and the recurrence.
20	Abdelgawad 2013 (22)	USA	Case report	2	3	Infantile	II	Lateral tension band plate (eight plate) and distal tibial external rotation osteotomy	1	this combination as the method of treatment for early stages of Blount’s disease as it corrects both elements of the disease and in the same time avoids the complications of proximal tibial osteotomy.
21	Oto etal 2012 (23)	Turkey	Retrospective	6	13 (12–14)	Adolescent		Eight-plate (Orthofix) hemiepiph-ysiodesis	1.8(1-2.6)	Don’t recommend the use of a tension band plate hemiepiphyseodesis (eight-plate, Orthofix) to treat severe adolescent Blount’s disease in obese children.
22	Putzeys etal 2012 (24)	France	Case report	1	14	Late-Onset	VI	Triple proximal tibial osteotomy	1	Late-onset Blount’s disease can be treated by a one-time procedure with internal stabilisation devices.
23	Kawu etal 2011 (25)	Nigeria	Retrospective	86	9.7 ± 3.9	Unkown	IV	Wedge, dome or chevron osteotomy	2	Postoperative MDA is a good outcome measure for surgical treatment of Blount disease and surgical correction should aim at producing post MDA ≤10°.

## Discussion

Blount’s disease characteristically induces progressive and three-dimensional deformities, such as proximal tibial varus, flexion, and internal rotation (26). Prolonged, untreated, or recurrent varus deformity increases the risk of degenerative osteoarthritis. Nonetheless, the pathogenesis of Blount’s disease remains unclear. Potential contributing factors encompass patient ethnicity, excessive knee joint pressure due to obesity or early weight-bearing, congenital varus development, nutritional status, genetics, histology, and intra-articular alterations (27).

Treatment approaches for Blount’s disease aim not only at correcting limb deformities but also restoring lower limb alignment, balancing leg length disparities, and ensuring post-maturity bone stability. The choice of treatment timing and mode is typically predicated on patient classification and deformity severity.

Clinical manifestations of infantile Blount’s disease (≤4 years old) often range from proximal tibial varus deformity and increased internal tibial rotation to notable “beak protrusion” of the medial epiphysis and metaphysis of the proximal tibia, and leg length discrepancy. Blount’s disease can be diagnosed when the metaphyseal-diaphyseal angle (MDA) of the affected limb is >16° in conjunction with corresponding physical changes (28). In the reported case, the patient displayed unilateral knee varus deformity around the age of two. Originally misdiagnosed with rickets at a primary care hospital. The X-ray of the patient’s knee joint before the second operation showed varus deformity of the left knee joint, sharp varus angulation of the metaphysis, and beak-like changes, MDA:19°. There were no skeletal deformities such as chicken breast and square skull, so rickets was not considered.The subsequent progression led to the identification of the condition as Blount’s disease.

Blount’s disease in infancy, characterized by epiphyseal-metaphyseal distortion, can be stratified into six stages according to Langenskiöld’s classification, primarily based on imaging results (29). Early intervention may prove beneficial for these patients, specifically with the application of orthopedic braces. Existing literature suggests that patients aged less than three years and those classified within Langenskiöld stage III or below may experience advantages from the extended use of anti-varus orthopedic braces (30). The knee ankle foot orthosis (KAFO) and the ankle foot orthosis (AFO) are the commonly chosen braces (31). Consequently, an early diagnosis and prompt initiation of brace treatment could potentially curtail the progression of the deformity, or even avert the necessity for subsequent surgical intervention.

Initial studies have identified certain risk factors contributing to the failure of conservative treatment, including obesity (body weight >90%), varus thrust, patient age (>3 years at treatment onset), bilateral involvement, and severe illness (Langenskiöld stage >III). For children under four with high-risk factors like high body mass index, persistent abnormalities following conservative treatment, or progressive worsening, osteotomies are necessary. These interventions can yield a total recovery rate of up to 80% (32, 33). Miraj (14) introduced a novel osteotomy approach termed the “Stepcut V” osteotomy, which has proven effective for patients under four with moderate to high deformities. The patient’s age at the time of the osteotomy is significantly associated with the recurrence rate. A delay in treatment until after age four can notably increase the recurrence rate (34, 35), with long-term recurrence rates ranging between 55% and 88% (25). These findings underscore the efficacy of early surgical intervention in controlling disease recurrence. Notably, there is no significant difference in the sagittal sequence among patients who underwent different osteotomy methods (11), eliminating the need for deliberate correction toward a valgus state, which does not affect the long-term recurrence (21). However, when the disease progresses to Langenskiöld stages V or VI, characterized by medial tibial plateau depression or medial tibial bone growth stagnation, osteotomy alone will not rectify the issue. It might lead to unequal lower limb length, exacerbating the deformities. Even with repeated osteotomy, deformities can recur before bone maturity. Successful interventions have been reported for Langenskiöld stage >IV patients under seven years of age, where epiphysiolysis of the proximal medial tibia combined with valgus osteotomy resulted in an over 80% success rate (36). An isolated case reported success with guided growth and hemiplateau elevation in treating advanced Blount’s disease, correcting the varus deformity, and preserving the affected limb’s growth potential. Postoperative strategies to manage patients’ BMI can further reduce the knee joint burden and the probability of recurrence (20). Therefore, for infantile-type patients unresponsive to ongoing conservative treatment or presenting high failure risk factors, timely osteotomy and orthopedic surgery can significantly improve prognosis. Alternative treatments for early-onset cases like growth modulation, hemiplateau elevation, and guided growth with hemiplateau elevation, despite their potential, have not been extensively studied, presenting various complications, uncertain outcomes, and limitations. These are currently subjects of debate within the field (21, 37, 38), yet their use highlights the significance of early intervention.

Late-onset patients with knee varus deformity, typically aged between 4 and 10 years, can be classified as having Juvenile Blount’s disease. This group’s specific treatment is contentious due to growth spurts and early growth plate fusion occurring between 6 and 8 years of age (32), and is usually discussed in conjunction with patients who develop the disease post age 10.

Blount’s disease occurring after the age of 10 is classified as Adolescent Blount’s disease. Roughly one third of varus deformities may be attributed to the distal femur (26), and the incidence is high among Caribbean African populations, most of whom are obese. Conservative treatment is generally deemed ineffective, which is strongly associated with patient age and size. Therefore, in order to fully correct deformities and prevent or delay the onset of osteoarthritis, surgical intervention, including osteotomy and hemiepiphyseodeses, is often considered.

Hemiepiphyseodeses and guided growth systems, known for their minimal invasiveness, target and correct deformities at the epiphyseal level. This correction process leverages the growth of the remaining healthy epiphysis while curbing the growth rate of the lateral epiphysis to achieve angular rectification. Barry et al. (13) suggested that lateral tibial hemiepiphyseodeses, based on a multicenter retrospective analysis, serve as a successful primary therapy for Blount’s disease. Adolescent Blount’s disease, particularly in those over 10 years old, is more likely to achieve full correction. Conversely, this treatment exhibits less effectiveness in infantile/juvenile Blount’s disease. The success rate of this intervention is reported to range from 50% to 88% (39), denoting considerable variance. A literature review posits that tibial hemiepiphyseodeses are most suitable for younger patients with mild varus deformity and less severe obesity, including those with a BMI below 40 kg/m2, body weight less than 100 kg, and varus deformity under 15° (18). Literature also suggests that hemiepiphyseodeses procedures should be considered when the patient’s growth potential is ensured for a minimum of 2–4 years, and the medial epiphyseal plate is still open and functional. Even if a recurrence transpires, remedial measures remain accessible (40, 41). In recent years, the tension band plate (TBP) has been predominantly utilized in surgery, considerably reducing the risk of postoperative complications compared to previously used transphyseal screws or staples (25, 42). This approach does not damage the original bone structure, ensuring minimal invasiveness. If orthopedic failure occurs, osteotomy can be selected as the final resort.

Osteotomy represents a definitive surgical intervention aimed at rectifying patients’ deformities, encompassing both acute and gradual corrective procedures. This surgical technique is particularly effective in addressing three-dimensional deformities in the lower extremities. Various osteotomy methods exist including open-wedge, close-wedge, oblique, dome, serrated, and inclined osteotomies, each boasting unique advantages (7). Notably, the prognosis for patients is relatively consistent across these methods. Gradual correction via osteotomy has been found to provide superior correction effects with fewer complications as compared to acute correction (43). Techniques such as the mono-lateral L-shaped lock, the Ilizarov apparatus, and Taylor Spatial Frame (TSF) are employed for external fixation, allowing for the gradual adjustment and restoration of normal lower limb anatomy. Despite extending the healing time, this method reduces the incidence of complications such as common peroneal nerve paralysis and osteofascial compartment syndrome (44). Previous studies suggest that acute correction is the preferred method when tibial varus is less than 15°, due to its lower trauma and cost implications. For more severe cases, gradual correction via external fixation is recommended. The most recent research underscores the relevance of bone maturity in patients with Blount’s disease in informing surgical correction strategies. The discrepancy between bone age and chronological age narrows with growth, impacting the severity of lower limb deformities and the necessary corrective measures. A regression study found that the bone age in 33 children with Blount’s disease was advanced by an average of 16 months (26 months in the early-onset group and 10 months in the late-onset group) (45). Therefore, a re-evaluation of preoperative bone age should be considered when treating children with Blount’s disease to facilitate the planning of an appropriate surgical technique, ultimately enabling effective deformity correction. At present, the study does have some limitations, the correction of deformity is mostly evaluated from both coronal and sagittal directions, and the adjustment of tibial rotation deformity has not been clearly studied. At present, there are some limitations in the study. The correction of deformity is mostly evaluated from both coronal and sagittal directions, while the adjustment of tibial rotation deformity has not been clearly studied.

The patient of this study was not given a clear diagnosis at the time of the initial visit. If orthopedic braces or necessary surgical treatment had been given in time, a one-time clinical cure might have been achieved. The patient first underwent an osteotomy at the age of seven. However, a recurrence occurred a year post-operation, necessitating a second internal fixation osteotomy at a different hospital. Despite this, the patient still exhibited a certain degree of varus deformity. The patient underwent several surgical procedures during this time, but postoperative recurrence was perhaps unavoidable due to the potential for continued growth of the patient’s unclosed epiphyseal plate. By the age of 20, when the epiphyses were fully closed and the bones fully matured, a final external fixation osteotomy was performed to achieve complete correction. Therefore, within this developmental stage from rapid growth to epiphyseal maturation, a sequence of traditional osteotomies and fixations appears unable to achieve desired surgical outcomes, often resulting in relapse or incomplete orthopedic results. This process can substantially increase both the physical trauma and financial burden on patients, indicating that traditional osteotomy may not be an ideal choice during this stage of patient growth and development, especially when the epiphysis has not yet closed.

## Conclusion

Consequently, the anomalous proliferation rate of the medial and lateral epiphyses in Blount’s disease patients invariably results in recurrent tibial varus. Thus, in early-onset Blount’s disease, prompt detection and treatment are paramount: osteotomy should be administered as soon as practicable when non-invasive treatments prove ineffective in patients below four years old, and a combined surgical approach should be timely considered in cases that are at or above Langenskiöld stage III. For patients who are older than four years or fall within the juvenile age range (4–10 years) but exhibit negligible growth potential, a single-instance osteotomy is performed. Adolescents (>10 years) with considerable growth potential are recommended to undergo hemiepiphysiodesis in conjunction with osteotomy upon maturity of epiphyseal closure (**Table 2**).

**Table 2:** Results in the literature.

Typing		Time of diagnosis	Stage	Treatment
Early-onset		<4years	≤III	Conservative treatment/Osteotomy
			>III	Hardware removal/Hemiplateau Elevation etal+ Osteotomy
		>4years		Surgical intervention is not recommended, When there is no growth potential, one-time osteotomy is feasible
Late-onset	Juvenile(4-10years)			There is no definite treatment plan, and the same as above is recommended
	Adolescent(>10years)	Growth potential≥2years		Hemiepiphyseodesis
		Growth potential<2years	Tibial varus <15°	Acute corrective osteotomy
			Tibial varus >15°	Gradual corrective osteotomy

## Data Availability

The original contributions presented in the study are included in the article/**Supplementary Material**. Further inquiries can be directed to the corresponding author.
