# Supplementary figures and images for: Case Report: Surgical timing for Blount’s disease: a case report and systematic review
Source: Front Endocrinol (Lausanne). 2025 Apr 9;16:1547679. doi: 10.3389/fendo.2025.1547679 (PMC12014453; doi:10.3389/fendo.2025.1547679)

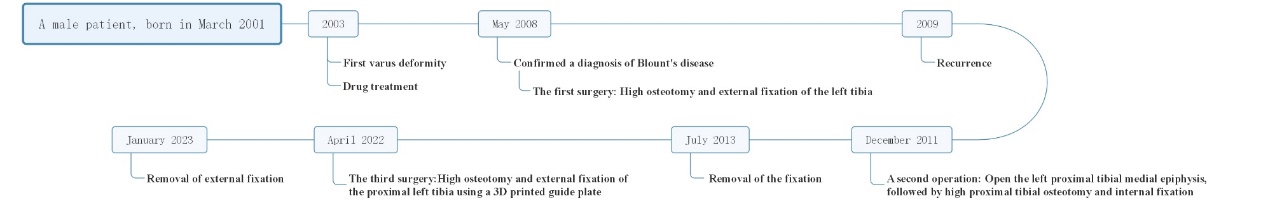


A FIGURE OR TABLE SHOWCASING A TIMELINE WITH RELEVANT DATA FROM THE EPISODE OF CARE

Supplement: Supplementary file 1 [file DataSheet1.docx]
